# Hearing Aid Amplification Improves Postural Control for Older Adults With Hearing Loss When Other Sensory Cues Are Impoverished

**DOI:** 10.1177/23312165241232219

**Published:** 2024-02-15

**Authors:** L. Behtani, D. Paromov, K. Moïn-Darbari, MS Houde, BA Bacon, M. Maheu, T. Leroux, F. Champoux

**Affiliations:** 1 School of Speech Language Pathology and Audiology, Faculty of Medicine, Université de Montréal, Montréal, QC, Canada; 2 Centre de Recherche de L’Institut Universitaire de Gériatrie de Montréal (CRIUGM), Montréal, QC, Canada; 3 Department of Psychology, University of British Columbia, Vancouver, BC, Canada; 4 Institut Universitaire Sur la Réadaptation en Déficience Physique de Montréal (IURDPM), Pavillon Laurier, CIUSSS du Centre-Sud-de-l’Île-de-Montréal, Montréal, QC, Canada

**Keywords:** aging, balance, falls, hearing aids, hearing loss

## Abstract

Recent studies suggest that sound amplification via hearing aids can improve postural control in adults with hearing impairments. Unfortunately, only a few studies used well-defined posturography measures to assess balance in adults with hearing loss with and without their hearing aids. Of these, only two examined postural control specifically in the elderly with hearing loss. The present study examined the impact of hearing aid use on postural control during various sensory perturbations in older adults with age-related hearing loss. Thirty individuals with age-related hearing impairments and using hearing aids bilaterally were tested. Participants were asked to perform a modified clinical sensory integration in balance test on a force platform with and without hearing aids. The experiment was conducted in the presence of a broadband noise ranging from 0.1 to 4 kHz presented through a loudspeaker. As expected, hearing aid use had a beneficial impact on postural control, but only when visual and somatosensory inputs were both reduced. Data also suggest that hearing aid use decreases the dependence on somatosensory input for maintaining postural control. This finding can be of particular importance in older adults considering the reduction of tactile and proprioceptive sensitivity and acuity often associated with aging. These results provide an additional argument for encouraging early hearing aid fitting for people with hearing loss.

## Introduction

Postural control requires the integration of visual, somatosensory and vestibular systems in order to maintain balance (De Sousa et al., 2012). In this process, acoustic cues can also act as auditory landmarks, aiding in the spatial positioning of the body in space in interaction with visual, vestibular, and proprioceptive inputs (Campos et al., 2018). In the case of hearing loss, participants may struggle to hear environmental sounds, elevating listening effort and reducing resources for balance control (Pichora-Fuller et al., 2016), which may be at play regarding the increased risk of falls among older adults (Lin & Ferrucci, 2012).

Given the role of hearing for postural control, the potential impact of hearing aids in restoring the postural control skills of individuals with hearing loss is of interest, especially for individuals with age-related hearing loss (ARHL). ARHL is the third most prevalent health problem in individuals over the age of 40, following arthritis and hypertension (Cruickshanks et al., 1998; De Sousa et al., 2009). The risk of falls also increases with age (e.g., Iwasaki & Yamasoba, 2014), which is known to have a significant impact on healthcare systems (World Health Organization, 2007). Falls represent the second leading cause of injuries and deaths among older adults (Jehu et al., 2020). Considering the increased risk of falls associated with hearing loss, the aging population, and the growing number of individuals with varying levels of hearing loss, the possibility that hearing aid use could enhance postural control and decrease the risk of falls is important. One explanation for the increased risk of falls in individuals with ARHL is related to sensory reweighting, a process that better maintains posture when sensory information from one modality is disturbed (Pasma et al., 2015). During reweighting, a feedback control model increases reliance on more consistent sensory modalities while decreasing reliance on the disturbed modality (Peterka, 2018). Recent data suggest that older individuals with ARHL may exhibit reduced balance capabilities due to a possible concomitantly reduction of the vestibular function and an increased dependence on somatosensory input for maintaining postural control when compared to individuals with normal hearing (Behtani et al., 2023). Given the deterioration of tactile and proprioceptive sensitivity and acuity associated with aging (De Sousa et al., 2009; Henry & Baudry, 2019; McIntyre et al., 2021), this increased reliance on an impaired system may not be effective for postural control (Behtani et al., 2023) and this, in part, may explain the increased risk of falls among the elderly (e.g., Iwasaki & Yamasoba, 2014; WHO, 2021).

A recent systematic review suggested that sound amplification via hearing aids could improve postural control in adults with hearing impairments (Mahafza et al., 2022). However, since only a few studies have been conducted and the results are still disputed, the authors emphasized that additional studies investigating the effect of hearing aids on postural control and balance in adults with hearing impairment are required.

Rumalla et al. (2015) were the first to examine the impact of hearing aid use on postural control in the elderly, although it was unclear whether the participants’ hearing loss was due to normal aging. This study of 14 individuals with hearing loss over the age of 65 years suggested that wearing hearing aids may improve static posture control. Based on an evaluation using the “Romberg on foam test,” the authors suggested that elderly individuals using hearing aids have better static postural ability when their hearing aids are turned on than when they are turned off in the presence of a point-source broadband white-noise sound (0–4 kHz). The specific characteristics of hearing loss and hearing amplification that they tested did not seem to have an impact on the results, perhaps due to the small sample size or the limits of the methodology.

Although tests not requiring specific equipment can be useful for diagnosing sensory-motor disorders in a clinical setting, they can only provide a gross indication of postural control efficiency (Paillard & Noé, 2015). To assess the impact of hearing aids on postural control, it is essential to use techniques allowing quantitative measurement of the center of pressure (CoP; Kalron & Achiron, 2013; Ostrowska et al., 2008; Piirtola & Era, 2006) and the contribution of different sensory information during postural control (Mahafza et al., 2022). 

Only four studies (Maheu et al., 2019; McDaniel et al., 2018; Negahban et al., 2017; Vitkovic et al., 2016) have used well-defined posturography measures to assess balance in adults with hearing loss with and without their hearing aids. Of these, only two examined postural control specifically in the elderly with hearing loss. Negahban et al. (2017) reported significant improvement in postural control when using hearing aids, with a significant correlation between postural control and the time of hearing aid acquisition: earlier acquisition was associated with better postural control. Contrary to these results, McDaniel et al. (2018) did not find a significant effect of hearing aid use on postural control. In all previous studies, the impact of hearing aid use in relation to sensory reliance was not examined. Therefore, the impact of hearing aid use on the dependency of the somatosensory system to maintain posture previously found in older adults with hearing loss (see Behtani et al., 2023) remains unclear.

The present study was aimed at assessing the impact of hearing aid use on postural control in older adults with ARHL using a method allowing for the examination of the reliance given to the visual and somatosensory systems during different conditions of sensory disturbance (Norré, 1993). As the benefit from hearing aid might improve with the time of use (e.g., Gantz & Turner, 2003; Lutz et al., 2008; Mosnier et al., 2009; Munro & Dees, 1996), it is of interest to distinguish between long-term effects (i.e., the benefit in postural control related to the time of acquisition) and short-term effects (i.e., hearing aids turned on or off). Hence, the study also aimed at examining performance in relation to the duration of hearing-aid use.

## Method

### Participants

Thirty individuals aged 40 and above took part in the study (15 women and 15 men, *M*_age_: 69 years, standard deviation (*SD*): 9). Participants were recruited from a database provided by the Research Center of the Institut Universitaire de Gériatrie de Montréal and the university clinic of the School of Speech-Language Pathology and Audiology of the Université de Montréal. All participants had ARHL diagnosed by an audiologist and were using hearing aids bilaterally (mean number of years of hearing aid use: 8 years, *SD*: 13). All participants self-reported that they were regular hearing aid users. Hearing thresholds were determined using an audiometer (Astera, GN Otometrics, Denmark). Pure-tone average hearing thresholds were determined as the mean across 0.25, 0.5, 1, 2, 3, 4, 6, and 8 kHz (average: 48 dB HL, SD: 13 dB).

Comprehensive peripheral vestibular assessment (for a complete description see: Maheu et al., 2015) included an evaluation of all six semicircular canals using the video head impulse test (vHIT; EyeSeeCam, Interacoustics, Taastrup, Denmark), an evaluation of both saccules with the cervical vestibular evoked myogenic potential (cVEMP; Eclipse EP-25/VEMP, Interacoustics, Taastrup, Denmark) and an evaluation of both utricles using the ocular vestibular evoked myogenic potential (oVEMP; Eclipse EP-25/VEMP, Interacoustics, Taastrup, Denmark). The cVEMP and oVEMP results were interpreted according to the presence or absence of a replicable wave using a 500 Hz tone burst at 95 dBnHL. If the waveform was absent or not replicable, the response was considered abnormal. For the vHIT, a vestibulo-ocular reflex gain between 1.0 and 0.8 was considered typical and less than 0.79 was considered abnormal. Incomplete evaluation (e.g., because of interference by eyelid movement, refusal to wear vHIT goggles due to discomfort, refusal to complete any task) or noisy results were deemed abnormal. Normal peripheral vestibular function was confirmed for only five participants.

### Procedure

Participants performed the modified clinical sensory integration in balance test (mCTSIB) on a force platform (Accusway, AMTI, USA) at a sampling rate of 50 Hz. During the test, a broadband pink noise (0.1–4 kHz) was presented through a loudspeaker placed one meter behind the participant (Sound Source Type 4224, Bruel & Kjaer, Denmark). The noise was adjusted to a comfortable level when using hearing aids for each participant, and this level was used for both the unaided and aided conditions. Each comfort level was 20–30 dB above the pure-tone average threshold.

Sway area and velocity were derived from recordings of the CoP and evaluated as described in previous studies (Maheu, Sharp, Landry, & Champoux, 2017; Maheu, Sharp, Pagé, & Champoux, 2017). The mCTSIB was used as it allows the isolation of different sensory components (i.e., vision, somatosensory, and vestibular) contributing to balance (Cohen et al., 1993). Participants stood in four different postural conditions: (a) eyes open on a firm surface; (b) eyes closed on a firm surface; (c) eyes open on foam (AIB Balance Foam, AIB, USA); (d) eyes closed on foam. Each trial lasted 60 s, during which the participant was requested to count backward starting from 1,000, as a cognitive task can be beneficial for postural control (Jehu et al., 2015). Participants were tested with and without hearing aids in a pseudorandom order. Each sensory condition was repeated 3 times and the median value in each condition was retained.

Two derived quantities were used to approximate sensory reliance (Norré, 1993). The role of visual information was evaluated by subtracting the sway parameters for Condition 1 (eyes open on the firm surface) from those for Condition 2 (eyes closed on the firm surface), and the role of somatosensory information was evaluated by subtracting the sway parameters for Condition 1 (eyes open on the firm surface) from those for Condition 3 (eyes open on the foam). These two quantities were calculated separately for sway area and sway velocity.

## Results

**Figure 1.**
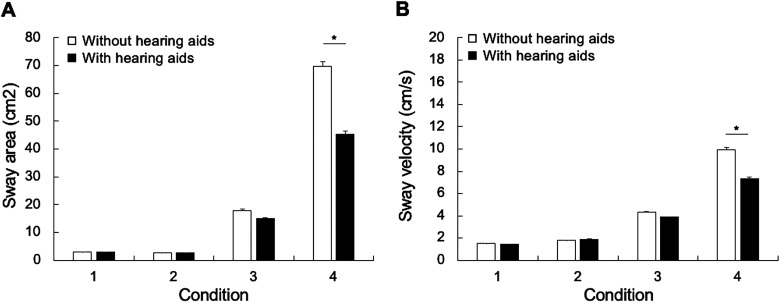
Average sway area (A) and sway velocity (B) without hearing aids (white bars) and with hearing aids (black bars) in four postural conditions in the presence of noise background: (1) eyes open on a firm surface; (2) eyes closed on a firm surface; (3) eyes open on foam; and (4) eyes closed on foam. The error bars represent the standard error of the mean. *p < .05.

Repeated measures analyses of variance (ANOVAs) were conducted for each aid use condition (with hearing aids; without hearing aids) × four postural conditions (conditions 1–4). For sway area, there was a significant effect of aid use, F(1, 54) = 5.098, *p* = .028, and a significant interaction between postural condition and aid use, F(1, 54) = 4.143, *p* = .007 (Figure 1(A)). For sway velocity, there was a significant effect of aid use, F(1, 54) = 5.736, *p* = .020, and a significant interaction between postural condition and aid use, F(1, 54) = 4.825, *p* = .032 (Figure 1(B)).

Independent *t*-tests using Bonferroni correction revealed a significant effect of hearing aid use for Condition 4 for both sway area, *t*(54) = 2.218, *p* < .001, and sway velocity, *t*(59) = 2.216, *p* < .001. No significant differences were observed for the other conditions. No significant correlation was found between the improvement in postural control with hearing aids and the degree of hearing loss (sway area: *r*(23) = .020, *p* = .928; sway velocity: *r*(23) = −.237, *p* = .276) or the duration of hearing aid use (sway area: *r*(23) = −.042, *p* = .849; sway velocity: *r*(23) = −.268, *p* = .217).

**Figure 2.**
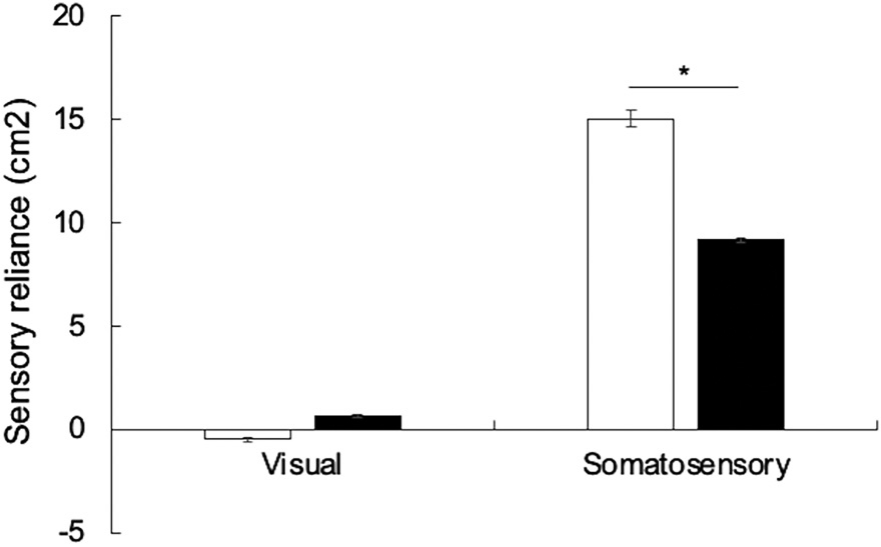
Sensory reliance for sway area without hearing aids (white bars) and with hearing aids (black bars). The error bars represent the standard error of the mean. *p < .05.

To examine sensory reliance with the use of hearing aids, repeated measures ANOVAs were conducted with the factors of hearing condition (with hearing aids; without hearing aids) and measures of sensory reliance (somatosensory; visual), separately for sway area and for sway velocity. For sway area, there was a significant effect of hearing aid condition, F(1, 58) = 5.771, *p* = .020, and a significant interaction between hearing condition and measure of sensory reliance, F(1, 58) = 4.764, *p* = .033 (Figure 2). Independent *t*-tests using Bonferroni correction showed no significant increase in reliance on the visual system, *t*(58) = 1.673, *p* = .031, but a significant decrease in reliance on the somatosensory system, *t*(59) = 17.532, *p* < .001 (Figure 2). For sway velocity, there was no significant effect of hearing aid condition, F(1, 58) = 0.135, *p* = .714, and no interaction, F(1, 58) = 4.866, *p* = .059.

## Discussion

Previous research has shown that auditory stimuli can improve postural control (Deviterne et al., 2005; Gandemer et al., 2017; Karim et al., 2018; Zhong & Yost, 2013). Our results are in accordance with previous studies suggesting that increasing the audibility of auditory cues through hearing aids may enhance spatial awareness and contribute to improved balance control (Maheu et al., 2019; Negahban et al., 2017; Rumalla et al., 2015; Vitkovic et al., 2016).

The present results also corroborate previous results suggesting that older individuals with ARHL exhibit increased somatosensory reliance relative to individuals with normal hearing (Behtani et al., 2023). Here, we additionally demonstrated that improving audibility in ARHL has an impact on such reliance on somatosensory cues. This aligns with previous studies reporting improvements in static balance due to hearing aids, particularly observed in postural conditions with challenging somatosensory input, such as balancing on a foam surface with eyes open or closed (Maheu et al., 2019; Negahban et al., 2017; Vitkovic et al., 2016).

One may wonder why such a change occurs in the somatosensory modality rather than in the visual modality. The most likely explanation is the presence of concomitant loss of vestibular sensitivity. It is well known that vestibular impairments can lead to an increased reliance on somatosensory cues to maintain posture (Okumura et al., 2015). Regardless of the likelihood of vestibular impairment for the majority of participants with ARHL, which can explain the increased reliance on somatosensory cues, the fact remains that using hearing aids has an effect on the sensory weight given to the somatosensory modality for postural control. Using hearing aids, reliance on somatosensory cues in participants with ARHL seems to be comparable to that of participants with normal hearing (see Behtani et al., 2023). The current data and those collected previously by our team (Behtani et al., 2023; Maheu et al., 2019), combined with the suggestion that the importance of the hearing modality is accentuated when other sensory inputs are poor or when task demands are high (Carpenter & Campos, 2020), suggest that such an improvement in postural control using hearing aids may only occur for those with both hearing and vestibular loss. Larger-scale studies aimed at very specifically evaluating people with ARHL and normal vestibular function compared to a group of identical size with impaired vestibular function would be necessary to assess the specific aspects of the improvements in postural control observed by wearing hearing aids.

Although our results are consistent with most previous research, they contrast with those of McDaniel et al. (2018), which did not find a significant effect of hearing aid use on postural control. The discrepancy may be related to their use of the Sensory Organization Test, which introduced a potential ceiling effect, a limitation admitted by the authors. Another potential explanation is related to the use of different auditory stimuli. Our study used broadband noise, whereas McDaniel et al. (2018) used multitalker babble. The varying types and perceptual significance of these auditory stimuli might exert distinct influences on balance. For instance, it is possible that broadband noise served as an auditory landmark, while speech functioned as a competing stimulus, demanding greater cognitive capacity in aging individuals (Bruce et al., 2019). The discrepancy may also be related to differences in participant characteristics, notably the vestibular status of the participants.

Caution should be exercised when considering the magnitude of the impact of hearing aids in relation to the severity of the type of hearing loss. It is also plausible that the residual auditory capabilities of some hearing-impaired individuals may be insufficient to provide auditory landmarks (Deviterne et al., 2005; Gandemer et al., 2017; Karim et al., 2018; Zhong & Yost, 2013) and, consequently, the use of hearing aids may not lead to substantial improvements in postural control. A more detailed examination of both the severity and specific type of hearing loss will be necessary to determine the influence of these variables on postural control.

This study provides further support for the immediate effects on postural control of switching hearing aids on and off, in line with previous studies (e.g., Negahban et al., 2017). The immediate impact observed on postural control and sensory reliance when activating hearing aids can be attributed to the participants’ ability to perceive sounds in their surroundings as effective spatial orientation landmarks (Negahban et al., 2017). However, unlike previous data that suggested a positive correlation between the time of hearing aid acquisition and the degree of benefit from hearing aids (the difference between off-aided and on-aided conditions) on postural control, our study did not find a significant correlation. One potential explanation for this disparity is the composition of our study cohort. Participants were all experienced hearing aid users, while Negahban et al. (2017) included users with as little as 3 months of experience. The greatest improvement in postural control might be experienced soon after the acquisition of hearing aids. However, it is possible that improvements in postural control with hearing-aid use are gradual, as suggested by previous investigations (e.g., Gantz & Turner, 2003; Lutz et al., 2008; Mosnier et al., 2009; Munro & Dees, 1996). Longitudinal studies conducted in the same individuals are needed to fully determine the relationship between duration of use and improvements in postural control. Data logging within hearing aids could be used to more precisely monitor the use of hearing aids. The results could be used to distinguish between long-term effects (i.e., the benefit in postural control related to the time of acquisition) and short-term effects (i.e., hearing aids turned on or off).

One limitation of our study was that only five participants were confirmed to have normal peripheral vestibular function, highlighting the significant influence of age on various vestibular measures for adults aged over 40 (Agrawal et al., 2013, 2019; Maheu et al., 2015). Research specifically aimed at evaluating postural control for individuals with normal vestibular function and for those with age-related vestibular loss is needed. In the present study, incomplete, noisy or not replicable results were treated as “fail.” Since vestibular status may influence the results, it would be informative to obtain a clear diagnosis of vestibular function. Additionally, there is a need for future studies to ascertain whether a significant improvement of postural control when using hearing aids occurs for elderly individuals without vestibular dysfunction. The enhancement of postural control may be more pronounced for adults with hearing loss who also experience deterioration in vestibular function relative to those with normal vestibular function (Maheu et al., 2019; Vitkovic et al., 2016).

The risk of falls is a major problem among the elderly (Iwasaki & Yamasoba, 2014; WHO, 2021), and our results confirm that hearing health is an essential component to consider in regard to this problem. Hearing amplification is already encouraged to improve communication in ARHL, and the present results suggest that hearing aid amplification could also be recommended to reduce the risk of falls.
